# Experiences from dual genome next-generation sequencing panel testing for mitochondrial disorders: a comprehensive molecular diagnosis

**DOI:** 10.3389/fgene.2025.1488956

**Published:** 2025-03-05

**Authors:** Elizabeth Gorman, Hongzheng Dai, Yanming Feng, William James Craigen, David C. Y. Chen, Fan Xia, Linyan Meng, Pengfei Liu, Robert Rigobello, Arpita Neogi, Christine M. Eng, Yue Wang

**Affiliations:** ^1^ Baylor Genetics, Houston, TX, United States; ^2^ Department of Molecular and Human Genetics, Baylor College of Medicine, Houston, TX, United States

**Keywords:** mitochondria, NGS, dual-genome, heteroplasmy, functional group analysis

## Abstract

**Introduction:**

The molecular diagnosis of mitochondrial disorders is complicated by phenotypic variability, genetic heterogeneity, and the complexity of mitochondrial heteroplasmy. Next-generation sequencing (NGS) of the mitochondrial genome in combination with a targeted panel of nuclear genes associated with mitochondrial disease provides the highest likelihood of obtaining a comprehensive molecular diagnosis. To assess the clinical utility of this approach, we describe the results from a retrospective review of patients having dual genome panel testing for mitochondrial disease.

**Methods:**

Dual genome panel testing by NGS was performed on a cohort of 1,509 unrelated affected individuals with suspected mitochondrial disorders. This test included 163 nuclear genes associated with mitochondrial diseases and the entire mitochondrial genome. A retrospective review was performed to evaluate diagnostic yield, disease-gene contributions, and heteroplasmy levels of pathogenic/likely pathogenic (P/LP) mitochondrial DNA (mtDNA) variants.

**Results:**

The overall diagnostic yield was 14.6%, with 7.7% from the nuclear genome and 6.9% from the mtDNA genome. P/LP variants in nuclear genes were enriched in both well-established genes (e.g., *POLG*) and more recently described genes (e.g., *FBXL4*), highlighting the importance of keeping the panel design updated.

**Conclusion:**

Variants in nuclear and mitochondrial genomes equally contributed to a 14.6% diagnostic yield in this patient cohort. Dual genome NGS testing provides a comprehensive framework for diagnosing mitochondrial disorders, offering clinical utility that can be considered as first-tier approach compared to single genome testing. Characterizing disease-causing genes, variants, and mtDNA heteroplasmy enhances understanding of mitochondrial disorders. Testing alternative tissues can further increase diagnostic yield.

## Introduction

While individually rare, the overall worldwide incidence of all mitochondrial diseases is approximately one in every 5,000 live births (Plutino et al., 2018). Due to the vast genotypic and phenotypic heterogeneity of mitochondrial diseases, obtaining an accurate and prompt diagnosis is often quite challenging, especially at the molecular level. Part of this complexity arises from normal mitochondrial function being the product of both the nuclear and mitochondrial genomes (Abadie, 2024; Craven et al., 2017; Kendall, 2012). Furthermore, although there are more than one thousand nuclear genes that have been implicated in mitochondrial biology (Pagliarini et al., 2008), only a small fraction of genes have established disease associations (Online mendelian inheritance in man, OMIM®, 2025; Stenson et al., 2014). In addition to sequencing the mitochondrial genome, targeted panels for next-generation sequencing (NGS) of nuclear genes for mitochondrial disorders are often offered by diagnostic laboratories. Separate mitochondrial genome panels are also available commercially (Wong, 2013; McCormick et al., 2013). Various factors, including known clinical relevance, disease prevalence, and cost are taken into consideration during the design of these panels. Because of this, commercial dual genome panels can often vary by hundreds of genes or have varying coverage of included genes. The advantage of simultaneously analyzing both the mitochondrial genome and nuclear mitochondrial genes has been recognized for some time, however, this approach is not always the standard of care (Abicht et al., 2018; Bonnen et al., 2013). This, to our knowledge, is the largest systematic evaluation on the clinical utility of dual genome NGS panels in the diagnosis of mitochondrial disorders. Although interactions between nuclear genes and mitochondrial genes are necessary to maintain mitochondrial function, there has been no practical evaluation of the actual contributions of each genome to the etiology of mitochondrial disease at this large a scale.

In this report, we summarize our experience as a clinical diagnostic laboratory in performing mitochondrial and nuclear NGS testing on a cohort suspected of having mitochondrial disorders. Preliminary analysis of results from diagnosed cases suggests that both genomes contribute equally. We show that the dual-genome NGS testing approach provides a comprehensive tool for the diagnosis of mitochondrial diseases. To our knowledge, this is one of the largest systematic analysis where interrogation of the mitochondrial and nuclear genomes was performed simultaneously.

## Method and materials

A retrospective analysis was performed on de-identified clinical and genetic data from 1,509 proband-only samples from patients with suspected mitochondrial disorders submitted to our laboratory for Dual Genome NGS panel testing. The mitochondrial genome was evaluated for all 37 mitochondrial genes with a minimum sequencing depth of 2,000x and an average sequencing depth of greater than 20,000x depth to allow for heteroplasmy calls down to 1.5%. The nuclear genome was evaluated for 163 nuclear genes with a minimum sequencing depth of 20x and an average sequencing depth of approximately 200x (see Supplementary Table 1 for nuclear gene list). Sample types included blood (n = 1,184), muscle (n = 214), extracted DNA (n = 78), and other (n = 33). Samples with one or more variants classified as pathogenic (P) or likely pathogenic (LP), within the genes assessed were tabulated. Genes were grouped by function to identify which pathways were most or least affected in patients with mitochondrial disease. This study was performed in accordance with protocols approved by the institutional review board for Human Subject Research at Baylor College of Medicine. Informed consent was obtained for genetic testing performed in a Clinical Laboratory Improvement Amendments (CLIA) and College of American Pathologist (CAP) accredited laboratory.

## Results

The overall diagnostic yield from the dual genome panel test was 14.6% (n = 220/1509). Of the 220 cases with a molecular diagnosis, the etiology of 115 cases was solely attributed to nuclear gene mutations, and the etiology of 103 cases was solely attributed to mitochondrial DNA mutations (see Supplementary Tables 2–4 for variant details). Two cases carried a dual diagnosis involving both genomes (Table 1). The diagnostic yield from nuclear genome testing was 7.7% whereas the mitochondrial genome yield was 6.9%.

**TABLE 1:** Molecular diagnosis made by findings in dual genome test.

Total dual genome cases	Solved by nuclear genome variants only	Solved by mitochondrial genome small variations only	Solved by mitochondrial genome large deletions	Dual diagnosis in both genomes
1509	115	92	11	2

The 220 cases molecularly diagnosed through the dual genome panel test constituted 14.6% of all cases.

**FIGURE 1:**
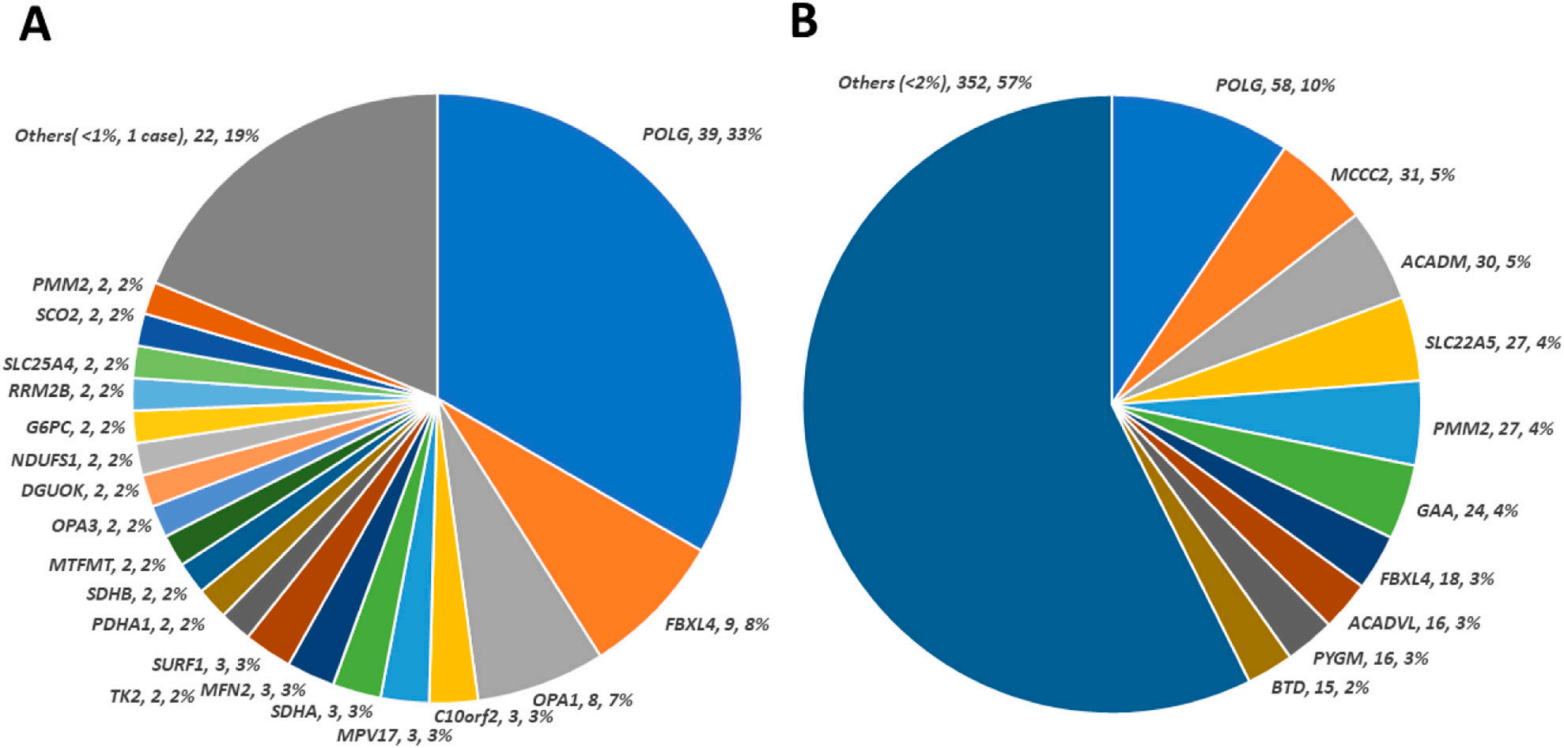
Contribution of observed nuclear genes/variants in patient cohort. **(A)** Distribution of causative defects in nuclear genes calculated from 117 solved cases. The size of the colored sectors represents the relative percentage of each causative nuclear gene. **(B)** Distribution of 614 unique pathogenic/likely pathogenic variants in nuclear genes regardless of causative (cases solved) or not (cases not solved) status. The size of the colored sector represents the relative percentage of unique pathogenic/likely pathogenic variant counts in genes annotated accordingly. There are 73 genes with a percentage of less than 1% of unique pathogenic/likely pathogenic variant, which are combined under “Others”.

The breakdown of nuclear genes implicated in solved cases is shown in Figure 1A. Most of these genes have been implicated in mitochondrial disease for more than 20 years. Most P/LP variants were in *POLG*, (n = 39, 33%). The second highest contributor to solved cases was *FBXL4* (Plutino et al., 2018), (n = 9, 8%), associated with mtDNA depletion syndrome 13. Among the solved cases, using functional groups, Mitochondrial DNA Maintenance, Expression, and Translation–Replication, maintenance, and transcription was the most enriched and Metabolism of cofactors–Biotin metabolism and Lipoic acid biosynthesis were the most scarce (Supplementary Table 5).

As shown in Figure 1B, 614 nuclear pathogenic or likely pathogenic (P/LP) variants were identified. The highest number of these variants was observed in *POLG* (Saneto and Naviaux, 2010). In the nuclear genome, P/LP variants from the top 10 genes account for almost half of the P/LP variants identified in our cohort. However, extensive genetic heterogeneity is present within this cohort which supports the utility of more extensive testing.

With the abundant variants and clinical cases collected, we also reviewed which functional pathways were primarily affected in patients with mitochondrial disorders. Grouped by function, (Stenton and Prokisch, 2020), there is the greatest enrichment of P/LP variants in Mitochondrial DNA maintenance, expression, and translation–Replication, maintenance, transcription genes and the most reduction in Mitochondrial dynamics, homeostasis, and quality control - Fusion genes (Supplementary Table 6).

**FIGURE 2:**
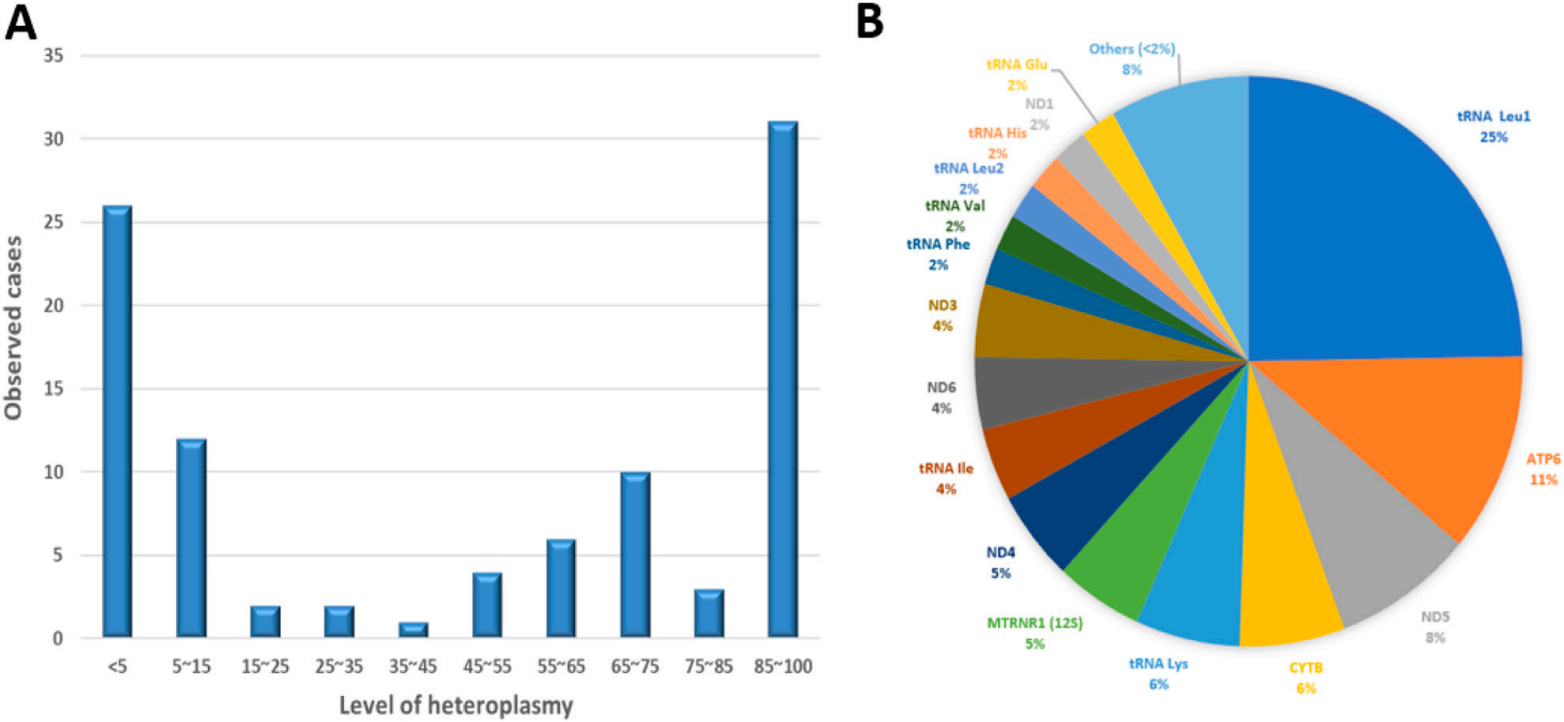
Observed defects in mitochondrial genome in the patient cohort. **(A)** Distribution of heteroplasmy level for observed pathogenic/likely pathogenic variants. Sample types were blood (78.5%), muscle (14.2%), extracted DNA (5.2%) and others (2.2%). **(B)** Distribution of pathogenic/likely pathogenic mitochondrial variants detected in our patient cohort. The size of the colored sectors represents the relative percentage of the number of pathogenic/likely pathogenic variants in genes annotated accordingly.

Mitochondrial variants were detected at various levels of heteroplasmy within different sample types. The mitochondrial P/LP variants appear to occur primarily at both high and low heteroplasmy in a bimodal distribution (Figure 2A). While there were recurrent variants in this cohort, there were no identifiable correlations between genotype and heteroplasmy compared to phenotype. In the mitochondrial genome, transfer RNA (tRNA) genes have the highest mutation rate (50%), although they comprise only 9% of the mitochondrial sequence (Anderson et al., 1981) (Supplementary Table 7). The common mutation, m.3243 A > G tRNA Leu (tRNA Leu1), was the most prevalent mitochondrial mutation in our cohort (Figure 2B).

In our cohort there are two cases that have pathogenic variants in both nuclear genes and mitochondrial genes. Patient 1 was diagnosed with developmental delay and muscle weakness in early childhood. This patient was found to carry a homozygous pathogenic variant in *TK2* and a m.13042G > A (p.A236T, *MT*-*ND5*) pathogenic variant at 3.1% heteroplasmy in a blood specimen. Patient 2 was a toddler with intractable refractory myoclonus/myoclonic seizures, hypotonia, lethargy and lactic acid peaks in basal ganglia. She was determined to have biallelic pathogenic variants in *POLG* and a homoplasmic m.11778G > A (p.R340H, *MT-ND4*) pathogenic variant, one of the common primary pathogenic variants associated with Leber Hereditary Optic Neuropathy (LHON).

Since the inheritance patterns are distinct for mtDNA-related disorders versus nuclear DNA-related disorders, these groups were examined for possible differences in age stratification. Our cohort showed meaningful age differences based on genomic etiology. The mean and median age at analysis for the group with nuclear gene defects are 12.4 and 3.2 years, respectively, while those for the group with mitochondrial DNA defects are 16.4 and 7.8 years, respectively (p-value = 0.013, two-tailed Mann-Whitney U test).

## Discussion

The most striking observation from our analysis is the nearly equal distribution of mitochondrial and nuclear gene defects among the solved cases in our cohort. A mitochondrial-only diagnostic rate of 6.7% has been reported over a 6-year period (Tang et al., 2013). A previous review found that the diagnostic rate for mitochondrial disease ranged from 8% to 24% when mtDNA was examined as a standalone test or as part of gene panels (Abicht et al., 2018). This finding emphasizes the importance of investigating both nuclear and mitochondrial genes concurrently. Simultaneous testing of the nuclear genome or exome in conjunction with high-read-depth mtDNA sequencing is often both expedient and cost-effective. Abicht et al. showed that high sequencing depth can identify low-level mtDNA heteroplasmy that would have been missed by standard WES analysis (Abicht et al., 2018). A dual diagnosis (nuclear and mitochondrial) could be missed if only one genome is assessed.

Our study also demonstrates the importance of updating panel design based on emerging literature. One gene, *FBXL4* was originally discovered through whole exome sequencing (Tang et al., 2013) and subsequently added to our panel. Since then, a significant number of *FBXL4* patients have been identified through our updated panel (Posey et al., 2017). *FBXL4* mutations contributed to an unexpectedly high fraction (9%) of solved cases. Since *FBXL4* dysfunction leads to an increase in mitochondrial fissioning and subsequent autophagy (Alsina et al., 2020) this suggests that the autophagic pathway is a major vulnerability of mitochondrial disease etiology. By extension, our study shows the importance of reanalysis by more comprehensive testing for capturing diagnoses from newly added genes like *FBXL4*. (Dai et al., 2017; Emperador et al., 2020; Liu et al., 2019). Additionally, the significant contribution of this gene to the etiology of mitochondrial disorders warrants further investigation on the mechanism of disease.

The most prevalent mitochondrial mutation, m.3243A > G in tRNA Leu, detected in about 1/4 of pathogenic variants in the mitochondrial genome in our cohort, aligns with the organelle control theory of mtDNA quality control. This theory suggests that selective degradation effectively targets mtDNA mutations in protein-coding genes but is less effective for mt-tRNA mutations (Kowald and Kirkwood, 2011).

The percentage of cases with a multi-locus molecular diagnosis (2/220) is lower than previously reported (Tang et al., 2013). This may be partially explained by the fact that patients in our cohort were analyzed by panel testing rather than by whole genome or exome sequencing (WGS/WES). Our patient cohort differs from those typically analyzed for mitochondrial disorders by WES, which may account for why certain genes are more frequently seen and diagnosed through our dual genome testing compared to WES. Additionally, our dual genome test is designed not only to identify mitochondrial disorders but also to identify metabolic disorders which may have a similar phenotype to those seen with mitochondrial disorders.

However, there were limitations to this study. In many cases, the clinical information was either very limited or not provided beyond a suspicion that the patient has a mitochondrial disease, making it difficult to establish correlations between genotypes and specific phenotypes.

Our nuclear panel size covers roughly one-tenth of the nuclear genes known to be involved in mitochondrial production and function. Although the exact role of many of these additional genes in causing mitochondrial disease may be unclear, a larger panel might increase the diagnostic yield in nuclear genes. Lastly, while the capture methodology of our nuclear gene panel offers much higher coverage of every base pair in the targeted regions, it is limited to exonic regions and ±20 bp of intronic borders.

This analysis highlights the benefits of comprehensive dual genome testing. In addition, it illustrates the need for frequent review of the literature and internal data to support continued improvements to this approach. Amending the gene list and including additional methodologies to target variants not captured adequately by NGS can further increase yield. While testing approaches combining WGS or WES with mitochondrial genome analysis have higher diagnostic yields, these approaches can be impeded by the lack of payor coverage and cost as well as varying levels of NGS coverage across different genes. Overall, the diagnostic yield for testing each genome individually was about half of the total diagnostic yield within our cohort. Our study highlights the clinical utility of a dual genome testing approach compared to single genome testing for evaluation of suspected mitochondrial disease.

## Data Availability

The datasets presented in this study can be found in online repositories. The names of the repository/repositories and accession number(s) can be found in the article/Supplementary Material.
